# Chemical Composition and Biological Activity of Essential Oil from *Dysphania ambrosioides* from Bulgaria

**DOI:** 10.3390/molecules31060946

**Published:** 2026-03-12

**Authors:** Andjelika Nacheva, Dimitar Bojilov, Stanimir Manolov, Iliyan Ivanov, Soleya Dagnon, Ivayla Dincheva, Neli Grozeva, Bogdan Goranov, Zlatka Ganeva

**Affiliations:** 1 Department of Organic Chemistry, Faculty of Chemistry, University of Plovdiv, 24 Tsar Assen Str., 4000 Plovdiv, Bulgaria; andjelikanacheva@gmail.com (A.N.); manolov@uni-plovdiv.bg (S.M.); iiiliyan@abv.bg (I.I.); solbono@abv.bg (S.D.); 2 Department of Agrobiotechnologies, AgroBioInstitute, Agricultural Academy, 1164 Sofia, Bulgaria; ivadincheva@abi.bg; 3 Faculty of Agriculture, Trakia University, Studentski Grad, 6000 Stara Zagora, Bulgaria; n.grozeva@trakia-uni.bg; 4 Department of Microbiology and Biotechnology, University of Food Technologies, 4002 Plovdiv, Bulgaria; b_goranov@uft-plovdiv.bg (B.G.); z_ganeva@uft-plovdiv.bg (Z.G.)

**Keywords:** essential oil, *Dysphania ambrosioides*, antioxidant activity (DPPH, HPSA, HRSA), inhibition of albumin denaturation (IAD), nonlinear logistic models, four-parameter logistic regression (4PL), five-parameter logistic regression (5PL)

## Abstract

In this article, we report a comprehensive analysis of the chemical composition and biological activity of *Dysphania ambrosioides* essential oil (DA-EO) originating from Bulgaria. Gas chromatography–mass spectrometry (GC–MS) analysis led to the identification of 53 constituents, revealing a complex phytochemical profile. The results classify the investigated oil as a thymol–carvacrol chemotype, dominated by oxygenated monoterpenes (56.79%), with thymol (19.45%) and carvacrol (14.30%) as the major components. This compositional profile differs markedly from the ascaridole-rich chemotypes commonly reported in the literature. The biological activity of DA-EO was evaluated through its antimicrobial, antioxidant, and anti-inflammatory properties. The oil exhibited broad-spectrum antimicrobial activity against pathogenic microorganisms such as *S. aureus*, *E. coli*, and *L. monocytogenes*. Antioxidant assays (HPSA, HRSA) indicated moderate activity, closely associated with the terpenoid composition of the oil. The anti-inflammatory potential, assessed via inhibition of albumin denaturation (IAD), was analyzed using nonlinear four-parameter (4PL) and five-parameter (5PL) logistic models. The obtained IC_50_ values (67.0–77.0 µg/mL) were comparable to those of the reference drug ibuprofen, highlighting the significant potential of DA-EO as a natural therapeutic agent.

## 1. Introduction

*Dysphania ambrosioides* (L.) Mosyakin & Clemants (synonym *Chenopodium ambrosioides* L.), a member of the family Amaranthaceae, is an aromatic medicinal plant with a wide geographical distribution, encompassing Central and South America, Africa, Asia, and Europe [1,2,3]. The species is characterized by a distinctive camphoraceous organoleptic profile, which is defined by the high content of monoterpenoids in its essential oil (EO) [4].

The yield of essential oil (EO) varies over a wide range, from 0.06% to 1.9% [4,5,6,7,8,9,10,11,12,13,14,15,16,17]. The chemical profile of DA-EO is characterized by considerable complexity and a high degree of variability, influenced by geographical origin, climatic conditions, and the phenological stage of the plant. A global comparative analysis of EO composition reveals the existence of distinct chemotypes. In North and South America, the predominance of specific terpenoid constituents has been shown to depend strongly on geographical location. In Mexico, an EO characterized by high levels of limonene (32.5%) and *trans*-pinocarveol (26.7%) has been reported [18]. In Brazil, variability is more pronounced: in Viçosa, an oil rich in (*Z*)-ascaridole (61.4%) was identified [15], whereas in Recife, chemotypes dominated by α-terpinolene (69.9%) [19] or α-terpinene (42.1%) [20] were described. In Lavras, the α-terpinene content is 40.7% [21]. In Cuba, chemotypes with high α-terpinyl acetate content (73.9%) [22], as well as those rich in carvacrol (62.4%) and ascaridole (47.1%), have been reported [23,24]. African EO samples are frequently characterized by elevated concentrations of α-terpinene. Among Nigerian samples, its content ranges between 48.7% and 63.1% [3,5,6,25]. For tropical regions such as Morocco, the composition of DA-EO varies substantially, with δ-3-carene (61.81%) identified as the major component [14]. Other studies from Morocco report α-terpinene as the predominant constituent, accounting for 61% of the essential oil [26]. In Benin, the α-terpinene content in DA-EO reaches 63.7% [12]. In contrast, in Togo, ascaridole has been identified as the major constituent (51.1%) [27]. In Asia, an exceptionally high degree of chemical diversity has been observed. In India, the major components vary between α-terpinene (up to 72.5%) [28,29,30] and ascaridole (40.7–44.9%) [13,31]. Chinese samples exhibit distinctive chemical profiles characterized by high levels of menthol (31.3%) [32], bornylene (42.6%) [33], or *p*-cymene (49.6%) [34]. In the Middle East (Iran), EOs with high concentrations of *cis*-ascaridole (43.4%) [4] and α-terpinyl acetate (73.9%) [35] have been identified.

The DA-EO exhibits pronounced antibacterial and antifungal activity, associated with oxygenated monoterpenes and peroxide compounds that disrupt membrane integrity, while the volatile fraction generally demonstrates higher activity than polar extracts [3]. Alitonou et al. reported that the EO showed stronger inhibitory activity against *Staphylococcus aureus* ATCC 25923 (MIC 1.56–1.71 mg/mL) compared to *Escherichia coli* ATCC 25922 (MIC 6.69–6.86 mg/mL). A bactericidal effect was observed only against *E*. *coli*, whereas a bacteriostatic action was suggested against *S. aureus* [12]. For *Dysphania ambrosioides*, MIC values of 256–1024 µg/mL against *S. aureus* and approximately 512 µg/mL against *Pseudomonas aeruginosa* have been reported [2], as well as the ability to modulate antibiotic resistance through inhibition of efflux pumps (TetK, NorA), leading to reduced MICs of co-administered antibiotics [2]. Limaverde et al. confirmed that the EO exerts a pronounced modulatory effect in multidrug-resistant *S. aureus* IS-58, despite the absence of a clinically significant direct antibacterial effect [36]. The antifungal activity includes inhibition of mycelial growth in the genera *Aspergillus*, *Fusarium*, *Colletotrichum*, *Macrophomina*, and *Verticillium* [2], as well as susceptibility of species from the genus *Candida* [37]. Ez-Zriouli demonstrated in vitro activity against *Staphylococcus aureus*, *Salmonella* sp., *Escherichia coli*, *Klebsiella pneumoniae*, and *Streptococcus* sp., with the exception of *Pseudomonas aeruginosa*. The highest sensitivity was observed in *Salmonella* sp., and the effect was associated with the predominance of α-terpinene, *p*-cymene, and ascaridole, as well as possible synergism among the components [38].

In traditional medicine, *D*. *ambrosioides* has been used for the treatment of intestinal parasites [39,40]. In America, the plant has been used as an effective anthelmintic treatment [41] and in cases of amoebic dysentery [42]. Extracts and the essential oil demonstrate anthelmintic activity against *Ancylostoma* spp. and *Haemonchus contortus* [43,44,45]. In Brazil and Mexico (where it is known as “epazote”), the plant is traditionally employed to treat parasitic infections caused by *Ascaris lumbricoides* and *Giardia lamblia*. In addition, this species has also been deployed for the ailment of cutaneous leishmaniasis in Brazil [46], and stomach ache and cold in Africa and Mexico [47,48]. *D*. *ambrosioides* is also used as an anti-inflammatory agent [3], a diuretic, and in the treatment of dermatomycoses [2,3]. In the Philippines, tribal people used leaves for the treatment of dyspepsia in children over the past century [2]. The decoction of leaves has also been utilized in Central America as an antispasmodic and to treat ulcers [49]. The EO also has potential as a therapeutic agent against dysentery [50]. Recent studies confirm the broad pharmacological profile of *D*. *ambrosioides*, encompassing antioxidant [1] and antiarthritic properties [51], as well as anti-nociceptive, sedative, and anti-inflammatory effects [1,52,53]. These activities are linked to the modulation of immune and inflammatory pathways and have been associated with soft tissue and bone repair [54], antitumor effects [55], analgesic action [56], anti-inflammatory and antinociceptive responses [1], and activity against *Helicobacter pylori* [57]. The essential oil exhibits pronounced antiprotozoal activity against *Leishmania amazonensis* [58,59], *Plasmodium falciparum* [60], and *Trypanosoma cruzi* [61]. The EO also exhibits negligible cytotoxicity toward the fungus *Candida albicans* [62].

Mechanistically, the antiprotozoal and cytotoxic effects are attributed to ascaridole, caryophyllene oxide, and carvacrol, which induce mitochondrial damage [63,64]. The endoperoxide bridge of ascaridole generates Fe^2+^-dependent free radicals, leading to lipid peroxidation and inhibition of mitochondrial complex I, thereby explaining both its pharmacological activity and toxicity [64]. In the context of cytotoxic and pharmacological potential, the essential oil demonstrates activity against human breast carcinoma cells (MCF-7) and other tumour cell lines such as acute B lymphoblastic leukemia (NALM6 and B15), myeloid leukaemia (K562), and Burkitt’s lymphoma (RAJI) (INCA 2011) [65,66], alongside reported preservative, hepato- and nephroprotective properties [67,68]. The species and its EO are also utilized in gastrointestinal disorders and in cosmetic applications as fragrance components [48,69].

Despite the global interest in *D. ambrosioides*, studies on the essential oil obtained from wild-growing populations in Bulgaria are currently lacking. The specific pedoclimatic conditions of the region exert a decisive influence on plant secondary metabolism, suggesting a unique chemical composition and potentially distinct pharmacological efficacy of the local resource.

The aim of the present study is to conduct a comprehensive characterization of the chemical composition of the essential oil extracted from *D. ambrosioides* and to evaluate its biological potential through a series of pharmacological assays. These include antioxidant, antimicrobial, and in vitro anti-inflammatory activities. To this end, modern statistical approaches for dose–response relationship analysis are applied, including linear regression as well as nonlinear four- and five-parameter logistic regression models (4PL and 5PL), enabling precise calculation of half-maximal inhibitory concentration (IC_50_) values and identification of the most appropriate model for describing the observed biological effects.

## 2. Results and Discussion

### 2.1. Analysis of DA-EO by GC-MS

The chemical composition of *D. ambrosioides* essential oil (DA-EO) was analyzed by gas chromatography–mass spectrometry (GC–MS). A total of 53 constituents were identified, revealing a complex phytochemical profile (Table 1, Figure S1).

The identification of the compounds was achieved through a comparative analysis of experimentally determined retention indices (RI), calculated relative to a homologous series of *n*-alkanes (C_8_–C_40_), and mass spectral data from the NIST 08 library, ensuring a high level of analytical reliability.

The literature reports substantial variations in the yield of DA-EO depending on the geographical origin and plant material used [5,6,7,8,9,10,11,12,13,14,15,16]. In the present study, the yield of DA-EO was 0.05% (*w*/*v*), which is among the lowest values reported to date. A comparable yield was described for Nigerian leaves (0.06%) [5], whereas most studies report considerably higher values, including 0.17–0.24% for Indian samples [6,7], 0.10–0.16% for material from China [11], 0.28% (wild) and 0.16% (cultivated) plants from Uttarakhand, India [10], 0.30% for Rwanda and Brazil [7,15], and 0.52% for Yemen [8]. Even higher yields have been documented for Moroccan material (0.75% in leaves and 1.2% in inflorescences) [14] and for samples from Benin (0.3–1.2%, *m*/*m*) [12]. These differences likely reflect the influence of climatic conditions, geographical factors, plant part (leaves vs. inflorescences or whole aerial parts), phenological stage, and possible chemotypic variability.

**Figure 1:**
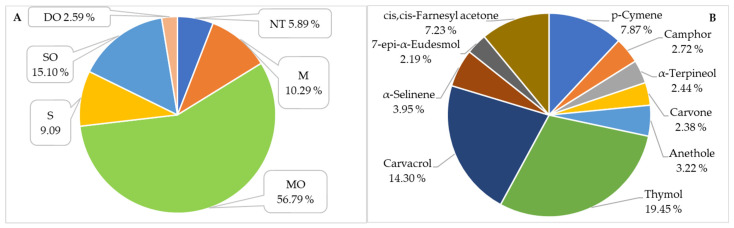
(**A**) Distribution of the main classes of terpene compounds in DA-EO; (**B**) main components of DA-EO. S-Sesquiterpene hydrocarbons, M-Monoterpene hydrocarbons, MO-Oxygenated Monoterpenes, SO-Oxygenated Sesquiterpenes, DO-Oxygenated Diterpenes, NT-non-terpene compounds.

GC–MS analysis revealed that oxygenated monoterpenes (MO; 56.79%) constituted the dominant fraction of the oil composition (Figure 1A). These were followed by oxygenated sesquiterpenes (SO; 15.10%), monoterpene hydrocarbons (M; 10.29%), and sesquiterpene hydrocarbons (S; 9.09%). Oxygenated diterpenes (DO) and non-terpenoid compounds (NT) were present in minor amounts. This distribution is of particular relevance, as oxygenated terpenoids are closely associated with the biological activity of the oil, including its antimicrobial, antioxidant, and anti-inflammatory properties [6,8,12,15,17,71,72].

The major constituents of the investigated essential oil were the phenolic monoterpenes thymol (19.45%) and carvacrol (14.30%). Their quantitative predominance defines the chemical profile of the oil as a thymol–carvacrol chemotype. Substantial amounts of other terpenoid constituents were also detected, including *p*-cymene (7.87%), *cis*,*cis*-farnesyl acetone (7.23%), α-selinene (3.95%), anethole (3.22%), camphor (2.72%), α-terpineol (2.44%), and carvone (2.38%) (Table 1, Figure 1B). From the literature, two chemotypes are mainly established: rich in ascaridol [4,8,13,15,16,24,27,31,40,42,44,45] and rich in α-terpinene [3,5,6,7,9,10,11,12,17,19,20,21,22,25,26,28,29,30,35,36,38,50]. In addition, samples rich in limonene [18], menthol [32], bornylene [33] and δ-carene [14] are found. Based on these data, the species from Bulgaria differs from all previously reported results, which makes it a unique species, perhaps for the Balkan Peninsula.

### 2.2. Antimicrobial Activity of the DA-EO

The antimicrobial activity of pure EO and diluted (39.8 mg/mL and 1.9 mg/mL) DA-EO was evaluated. The results, presented in Table 2, demonstrate that the native EO exhibits an inhibitory effect against all tested pathogenic microorganisms.

The data indicate that the susceptibility of the isolates is strain-specific. A high degree of sensitivity to DA-EO was observed among *Staphylococcus aureus* species. Strain *S. aureus* ATCC 25923 exhibited zones of inhibition for both the pure EO (28.00 ± 0.71 mm) and the diluted oil with a concentration of 39.8 mg/mL (13.00 ± 0.71 mm). In contrast, antibacterial activity for *S. aureus* ATCC 6538 was recorded only with the application of pure EO, resulting in an inhibition zone of 11.00 ± 0.71 mm. These findings indicate that DA-EO may represent a promising candidate for further investigation as a potential alternative antimicrobial agent against *S. aureus* strains. The higher susceptibility of *S. aureus* compared to Gram-negative isolates is consistent with the known increased resistance of Gram-negative bacteria due to the presence of an outer membrane barrier. These findings suggest that DA-EO has potential for use as an alternative agent for controlling antibiotic-resistant *S. aureus* strains. A similar trend was established for members of the genus *Escherichia coli*. Strain *E*. *coli* ATCC 8739 showed sensitivity to both the pure EO and EO with a concentration of 39.8 mg/mL, with inhibition zones of 15.00 ± 0.71 mm and 9.00 ± 0.00 mm, respectively. For *E. coli* ATCC 25922, a suppressive effect was observed only with the undiluted EO (10.00 ± 0.00 mm). The data in Table 2 confirm the antibacterial effect of DA-EO against *Salmonella enterica* ATCC 13076, with inhibition zones for the pure EO and the diluted oil with a concentration of 39.8 mg/mL, amounting to 16.00 ± 0.71 mm and 10.00 ± 0.71 mm, respectively. The growth of *Listeria monocytogenes* ATCC 19115 was affected solely by the undiluted essential oil (17.00 ± 0.71 mm). In conclusion, pure DA-EO demonstrates broad-spectrum antimicrobial activity against all investigated pathogens. The pronounced inhibitory activity, particularly against *S. aureus* ATCC 25923 (28.00 ± 0.71 mm), may be associated with the high proportion of phenolic monoterpenes identified in the GC–MS profile of DA-EO, which aligns with previously reported activities of thymol- and carvacrol-rich essential oils. Thymol has been shown to exert antibacterial and antifungal effects primarily through disruption of bacterial membrane integrity, increased permeability, and leakage of intracellular constituents, ultimately leading to cell death [73]. Similarly, carvacrol demonstrates strong antimicrobial activity against a broad spectrum of pathogenic and antibiotic-resistant strains, with membrane destabilization and cytoplasmic disintegration identified as major mechanisms of action [74]. Recent studies further confirm that thymol- and carvacrol-rich essential oils exhibit significant inhibitory activity, including measurable MIC values against clinically relevant bacteria [75,76]. Thyme oil and its major constituents have also demonstrated efficacy against MRSA and other resistant isolates, supporting their relevance as alternative antimicrobial agents [77]. Moreover, thymol-based formulations have been reported to retain strong antibacterial activity in applied systems, including food-related and biofilm-associated contexts [78,79]. Collectively, these data support the assumption that phenolic monoterpenes are key contributors to the antimicrobial efficacy observed for DA-EO in the present study.

### 2.3. Antioxidant Activity, Assessed by DPPH, HPSA, and HRSA

While Cu^2+^ and Fe^2+^ ions are essential cofactors in numerous enzymatic and physiological processes, their presence in a free state can induce significant oxidative damage. These cations catalyze the oxidation of ascorbic acid, generating reactive oxygen species (ROS), such as superoxide radicals (O_2_^•−^) and hydrogen peroxide (H_2_O_2_). Furthermore, through Fenton and Haber–Weiss reactions, Cu^2+^ and Fe^2+^ interact with H_2_O_2_ and organic hydroperoxides (ROOH), leading to the formation of highly reactive hydroxyl radicals (^•^OH) [80,81]. The resulting oxidative stress leads to the degradation of proteins, DNA, and essential phospholipids, contributing to the pathogenesis of diseases such as cancer, atherosclerosis, cardiovascular disorders, and Alzheimer’s disease [82]. This process is further exacerbated by inflammatory mechanisms, which stimulate ROS generation. Specifically, the formation of superoxide anions serves as a precursor for broader oxidative cascades, highlighting the critical link between inflammatory responses and cellular damage.

The antioxidant activity of DA-EO was evaluated using DPPH radical scavenging, hydrogen peroxide scavenging activity (HPSA), and hydroxyl radical scavenging activity (HRSA) assays. The obtained results were compared with those of ascorbic acid (AA) and quercetin (Qrc) and expressed as *IC*_50_ values (µg/mL) (Table 3).

In the DPPH assay, AA and Qrc exhibited high antioxidant activity (*IC*_50_ = 3.19 ± 0.03 and 4.98 ± 0.33 µg/mL, respectively). In contrast, DA-EO showed considerably weaker activity, with an *IC*_50_ of 2572 ± 113 µg/mL and a total antioxidant capacity of 24.71 ± 0.25 µM TE/g (Table 3).

In the HPSA assay, AA demonstrated the lowest *IC*_50_ value (24.84 ± 0.35 µg/mL), followed by Qrc (69.25 ± 1.82 µg/mL). DA-EO exhibited moderate hydrogen peroxide scavenging activity, with an *IC*_50_ of 163 ± 4 µg/mL (Table 3).

In the HRSA assay, DA-EO displayed an *IC*_50_ of 97.55 ± 1.37 µg/mL, indicating moderate activity against hydroxyl radicals, which is comparable to that of Qrc (70.11 ± 1.17 µg/mL) (Table 3).

The results indicate that the antioxidant activity of DA-EO is clearly dependent on the assay employed, with the weakest activity observed in the DPPH test and relatively higher activity in the HRSA assay. This behaviour can be explained by the chemical composition of the essential oil, as determined by GC–MS analysis.

DA-EO is dominated by oxygenated monoterpenes (MO; 56.79%), including thymol (19.45%), carvacrol (14.30%), camphor (2.72%), *α*-terpineol (2.44%), carvone (2.38%), and anethole (3.22%), as well as oxygenated sesquiterpenes (SO), such as *cis*,*cis*-farnesyl acetone (7.23%) and *7-epi-α*-eudesmol (2.19%), along with other terpenoid hydrocarbons. It is known that terpenoid compounds have a limited ability to donate hydrogen atoms or electrons, which is a key mechanism for neutralizing free radicals [83,84]. For this reason, DA-EO shows low effectiveness in the DPPH assay—a method based on the reduction in the stable DPPH^•^ radical [12]. The absence or low content of classical phenolic structures in the oil’s composition likely limits its radical-scavenging potential in this specific model. However, it should be noted that the DPPH assay primarily reflects a single mechanism of antioxidant action (radical scavenging through direct electron/hydrogen donation) and does not capture other possible mechanisms, such as metal ion chelation, modulation of enzymatic antioxidant systems, or inhibition of lipid peroxidation. In this context, it has been reported that ascaridole-rich oils can significantly enhance overall antioxidant capacity [8], likely through alternative mechanisms distinct from direct radical scavenging. This suggests that the antioxidant potential of DA-EO should not be evaluated solely based on DPPH results, but rather within a broader spectrum of methods and mechanistic approaches. The moderate activity of DA-EO in the HPSA assay, and particularly in the HRSA assay, suggests that certain oil components may participate in the neutralization of reactive oxygen species via alternative mechanisms, including peroxide decomposition or interaction with highly reactive radicals such as ^•^OH. In this context, oxygenated terpenoid compounds play a key role.

Comparison with AA and Qrc highlights the substantially lower antioxidant efficiency of DA-EO, which is expected given the absence of flavonoids and phenolic acids in essential oils. Nevertheless, the relatively good activity against hydroxyl radicals indicates that DA-EO may have potential as a supplementary antioxidant, especially in systems dominated by highly reactive oxygen radicals. The antioxidant profile of DA-EO is closely associated with its GC–MS-determined composition and reflects the predominance of terpenoid compounds exhibiting assay-specific antioxidant activity.

### 2.4. Anti-Inflammatory Activity Assessed by Inhibition of Albumin Denaturation (IAD)

Inflammation is a protective response of living tissues to various stimuli, including physical injury, thermal stress, microbial infections, or exposure to aggressive chemical agents. This cellular response leads to specific pathological manifestations, characterized by erythema, localized increase in temperature, edema, pain, and impaired physiological function. The inflammatory process is a key pathogenic factor in numerous diseases, including arthritis, stroke, and cancer. Protein denaturation plays a central role in initiating this response and is closely associated with the development of chronic inflammatory conditions [85]. According to Opie [86], tissue injury during life might be referable to denaturation of the protein constituents of cells or of intercellular substance. Hence, the ability of a substance to inhibit the denaturation of protein signifies apparent potential for anti-inflammatory activity.

**Figure 2:**
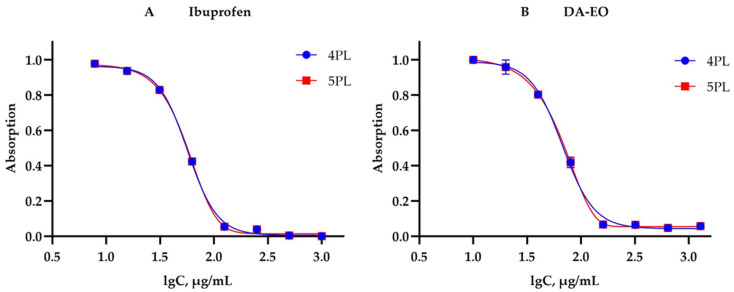
Dose–response curves for (**A**) ibuprofen and (**B**) *Dysphania ambrosioides* essential oil (DA-EO) obtained from the inhibition of albumin denaturation (IAD) assay. Experimental data (observed) and predicted values derived from four-parameter (4PL) and five-parameter (5PL) logistic models are shown, with *IC*_50_ values indicated. The data are presented as mean ± SD (n = 3).

The anti-inflammatory activity of DA-EO was evaluated through inhibition of albumin denaturation (IAD) and compared with the standard nonsteroidal anti-inflammatory drug ibuprofen. The data were analyzed using linear regression (LR) as well as parametric four-parameter (4PL) and five-parameter (5PL) logistic models (Table 4, Figure 2).

The 4PL and 5PL models demonstrated excellent agreement between the experimental and predicted values, with coefficients of determination (R^2^) ranging from 0.9967 to 0.9992 (Table 4). This confirms that nonlinear logistic models are more suitable for describing the sigmoidal dose–response relationship in the IAD assay compared to linear regression, which exhibited a considerably lower goodness of fit (R^2^ ≈ 0.88) (Table 4).

According to the 4PL model, the *IC*_50_ value for DA-EO was 67.0 µg/mL, which is very close to that of the reference drug ibuprofen (56.9 µg/mL). Using the 5PL model, the *IC*_50_ values shifted slightly (77 µg/mL for DA-EO and 64 µg/mL for ibuprofen), highlighting the importance of model selection in the interpretation of bioactivity (Table 4).

The Hill slope (***p***) is a fundamental parameter in logistic models for dose–response analysis (4PL and 5PL). It defines the steepness of the sigmoidal curve at the inflection point (*IC*_50_) and reflects the dynamics of the biological response to changes in concentration. The value of ***p*** provides information on the sensitivity of the tested system as well as the cooperativity of interactions between the analyte and the biological target [87]. In the context of the applied IAD assay, the Hill slope parameter (***p***) characterizes the intensity of albumin stabilization at increasing concentrations of ibuprofen and DA-EO (Table 4). The results of the present study show higher ***p*** values for ibuprofen (***p*** = 3.23–3.25) compared to DA-EO (***p*** = 2.95–3.10). The fact that ***p*** > 1 for both substances indicates positive cooperativity or a strongly synchronized effect, whereby minimal changes in concentration around the *IC*_50_ result in a sharp change in the biological response. The relatively lower Hill coefficient for DA-EO (compared to ibuprofen) reflects a more gradual increase in effect. This behaviour is likely due to the complex phytochemical matrices typical of essential oils, where multiple bioactive components may act additively or synergistically, but differences in their individual affinity toward the protein result in a more diffuse overall response.

Application of the 5PL model indicates pronounced asymmetry (***s***) in the experimental dose–response curves. The asymmetry parameter exceeds unity for both samples (***s*** = 1.33 for DA-EO and ***s*** = 1.29 for ibuprofen). This deviation from ideal symmetry (characteristic of the 4PL model, where ***s*** = 1) justifies the use of 5PL regression to achieve higher statistical adequacy [88,89].

The 5PL model allows for a more precise description of the inhibition kinetics, as it captures the specific behaviour of the system in the asymptotic regions, particularly at high inhibitor concentrations.

The results indicate that DA-EO exhibits significant in vitro anti-inflammatory activity, as assessed by the IAD assay. Although its potency is lower than that of ibuprofen, DA-EO demonstrates a clear, well-defined dose-dependent effect. The 4PL and 5PL logistic models provide a more reliable quantitative description of the activity compared to linear regression. Hill slope values (***p*** ≈ 3.0) indicate strong positive cooperativity in the inhibitory process, suggesting that the anti-denaturation effect is triggered sharply within a narrow concentration range, a pattern characteristic of highly specific interactions. These findings support the potential of DA-EO as a natural source of anti-inflammatory agents.

Analysis of published studies further shows that essential oils with a high thymol content exhibit anti-inflammatory activity in in vitro and in vivo models, including models of acute inflammation. A higher proportion of thymol correlates with a stronger biological effect. Direct experimental evidence also confirms the anti-inflammatory action of thymol as an individual compound, as it suppresses key inflammatory pathways, including NF-κB, MAPK, and arachidonic acid metabolism. In LPS-induced cellular models, thymol reduces NF-κB p65 phosphorylation and the expression of pro-inflammatory mediators, supporting its role as a principal bioactive component of thymol-rich essential oils [90].

Essential oil from *Thymus vulgaris*, rich in thymol and carvacrol, demonstrates significant anti-inflammatory activity in carrageenan-induced pleurisy in rats by reducing exudate formation and leukocyte migration [91]. Similarly, essential oil from *Satureja hortensis*, administered orally, inhibits approximately 60% of carrageenan-induced paw edema and is characterized by high levels of carvacrol and thymol [92]. In addition, carvacrol as an isolated compound exhibits anti-inflammatory activity in vivo, reducing histamine-induced paw edema and arachidonic acid-induced ear edema, likely through the modulation of inflammatory mediators [93].

Based on the presented experimental data, it can be concluded that DA-EO exhibits significant anti-inflammatory activity, characterized by a clear dose-dependent effect and mechanistic relevance related to the modulation of key inflammatory pathways. In combination with literature data on the anti-inflammatory properties of thymol- and carvacrol-containing essential oils, these findings support the consideration of DA-EO as a potential natural anti-inflammatory agent.

### 2.5. Statistical Evaluation and Selection of Regression Model

To determine the half-maximal inhibitory concentration (*IC*_50_) of DA-EO and ibuprofen, the experimental data were analyzed by comparing three mathematical models: linear regression (LR), four-parameter logistic (4PL), and five-parameter logistic (5PL). Model validation was performed using a set of statistical criteria, including the residual sum of squares (RSS), Akaike information criterion (AIC), Bayesian information criterion (BIC), and the adjusted coefficient of determination ($R_{ad}^{2}$) (Table 5).

The statistical parameters clearly indicate that nonlinear logistic models outperform linear regression in terms of precision and description of the biological response:

Adjusted coefficient of determination ($R_{ad}^{2}$): The 4PL and 5PL models demonstrated excellent agreement with the experimental data, with $R_{ad}^{2}$ values ranging from 0.9967 to 0.9992, whereas the linear model showed considerably lower correlation ($R_{ad}^{2}$ = 0.8740–0.9038) (Table 5).

Residual sum of squares (RSS): The RSS values for the logistic models (4PL and 5PL) ranged from 1.60 × 10^−3^ to 3.40 × 10^−3^, which is two orders of magnitude lower than those observed for LR (0.163–0.164), confirming minimal deviation of the observed points from the calculated curve (Table 5).

Model optimization via information criteria (AIC and BIC): When comparing 4PL and 5PL, the principle of parsimony was applied. Although the 5PL model accounts for the asymmetry parameter (***s*** = 1.29–1.33), the statistical criteria indicate 4PL as the most suitable model for this system:

- Information criteria: The 4PL model exhibited the lowest (most negative) AIC (−59.20 for ibuprofen and −54.11 for DA-EO) and BIC (−58.88 for ibuprofen and −53.79 for DA-EO) values (Table 5).
- *IC*_50_ precision: *IC*_50_ values calculated using the 4PL model (56.9 ± 1.2 µg/mL for ibuprofen and 67.0 ± 1.7 µg/mL for DA-EO) were associated with narrower confidence intervals and higher statistical significance compared to those obtained via the 5PL model (Table 4).

The AIC and BIC clearly indicate that the 4PL model is statistically preferred over the 5PL model for both samples. Although the 5PL model provides a marginal reduction in RSS, this improvement is insufficient to justify the additional asymmetry parameter, resulting in higher (less negative) AIC and BIC values. Therefore, the 4PL model provides the optimal balance between accuracy and parsimony for describing the IAD dose–response data.

## 3. Materials and Methods

### 3.1. Chemicals and Reagents

Chromatographic-grade methanol (VWR, Vienna, Austria) was used for HPLC analysis. Water for HPLC was obtained using a Millipore purifier (Millipore, Burlington, MA, USA). Urea, glycerol, ibuprofen, potassium dihydrogen phosphate, dipotassium hydrogen phosphate, sodium chloride, potassium chloride, sodium carbonate, ferrous sulphate, ferrous dichloride, hydrogen peroxide, ascorbic acid, quercetin, DPPH (2,2-diphenyl-1-picrylhydrazyl), and phenanthroline were supplied by Sigma-Aldrich Chemie GmbH, Buchs, SG, Switzerland. Human albumin 20%—BB, 200 g/L was purchased from BB-NCIPD Ltd., Sofia, Bulgaria.

### 3.2. Plant Material

The specimen of *D. ambrosioides* was collected in the village of Sinitovo, Bulgaria, in the period 2023–2024. The plants were gathered at the full flowering stage. The aerial parts of the plant were dried at room temperature in the absence of direct sunlight. The plant material was dried to a residual moisture content of 8–10%. Moisture content was determined in accordance with Ph. Eur. 7.0 (2.2.32 Loss on drying) using a moisture balance (MB45 Moisture Analyser, Florham Park, NJ, USA). The dried material was further ground into powder, packed into multilayer paper bags, and stored in a dark room at ambient temperature. The total amount of dry drug used to isolate the essential oil was 5 kg.

### 3.3. Obtaining Essential Oil

The essential oil was isolated from the dried aerial parts of *D. ambrosioides* via hydrodistillation using a Clevenger-type apparatus. The distillation was conducted at a plant material-to-water ratio of 1:10 (*w*/*v*), where 100 g of dried plant material was distilled in 1 L of distilled water. The extraction process lasted for 3 h, measured from the onset of boiling. This procedure was repeated a total of 31 times to ensure a sufficient quantity of essential oil for subsequent analyses. The resulting oil was dried over anhydrous sodium sulphate (Na_2_SO_4_) and stored in tightly sealed amber glass vials in a freezer until further use.

### 3.4. Analysis of DA-EO by GC-MS

The chemical composition of the essential oil was analyzed using gas chromatography–mass spectrometry (GC–MS) on an Agilent Technologies 7890A (Santa Clara, CA, USA) gas chromatograph coupled to a 5975C mass selective detector. Compound separation was achieved on a DB-5 ms capillary column (30 m × 0.25 mm i.d.). Helium was used as the carrier gas at a constant flow rate of 1.0 mL/min. The injector temperature was maintained at 250 °C, and samples (1.0 µL) were injected in split mode with a split ratio of 10:1.

The oven temperature program started at 40 °C and was held for 5 min, then increased at a rate of 5 °C/min to 300 °C, where it was maintained for 10 min. Mass spectra were recorded in electron ionization (EI) mode at 70 eV, with a scanning range of 50–550 *m*/*z*.

Retention indices (RI) of the detected compounds were calculated using a homologous series of *n*-alkanes (C_8_–C_40_, Sigma) analyzed under identical chromatographic conditions. Identification of EO constituents was performed by comparing calculated RI values and mass spectra with reference data from the Adams library and the NIST 2008 database (Adams, 2007; NIST, 2008).

Quantitative determination was performed by GC–FID using the same chromatographic column and temperature program as applied for GC–MS analysis. The injector and detector temperatures for GC–FID were set to 220 °C and 280 °C, respectively. Helium was used as the carrier gas at a flow rate of 1.0 mL/min, and the relative content of individual components was calculated using the peak area normalization method.

### 3.5. Antimicrobial Activity of DA-EO

The antimicrobial activity of DA-EO and its diluted solutions was evaluated against the following pathogenic microorganisms: *Escherichia coli* ATCC 8739, *Escherichia coli* ATCC 25922, *Staphylococcus aureus* ATCC 6538, *Staphylococcus aureus* ATCC 25923, *Salmonella enterica subsp. enterica serovar Enteritidis* ATCC 13076, and *L. monocytogenes* ATCC 19115. These pathogens are commonly encountered in food. Suspensions of the pathogenic microorganisms were prepared after 24 h of cultivation at 37 °C on LBG agar, adjusted to cell concentrations according to the McFarland standards: 0.5 (corresponding to 1.5 × 10^8^ CFU/mL) for Gram-positive strains and 1.0 (corresponding to 3.0 × 10^8^ CFU/mL) for Gram-negative strains. These suspensions were used to inoculate Petri dishes containing LBG agar.

After agar solidification, sterile paper discs (6 mm diameter) were placed on the surface, and 6 µL of the tested essential oil and its diluted solutions were applied onto each disc. The inoculated Petri dishes were then incubated at 37 °C. Antimicrobial activity was assessed after 48 h of incubation by measuring the zones of inhibition in millimetres (mm).

### 3.6. Antioxidant Activity of DA-EO

#### 3.6.1. DPPH Assay

The DPPH^•^ radical scavenging assay was performed according to the method described by Docheva et al. [94]. A freshly prepared DPPH^•^ solution with a concentration of 0.12 mM was used. Briefly, 2.0 mL of the DPPH^•^ solution was mixed with 2.0 mL of the test sample at various concentrations. The reaction mixtures were incubated in the dark for 30 min at room temperature. The absorbance was measured at 517 nm using a Camspec M508 spectrophotometer (Camspec Ltd., Leeds, UK). All measurements were carried out under dim light. The percentage of radical scavenging activity (RSA) was calculated relative to a control using the following equation:

(1) $$RSA,\;\%=\;\left[\frac{A_{o}-A_{S}}{A_{o}}\right]\times 100\;$$

where *Ao* is the absorbance of the control blank, and *A_S_* is the absorbance of the samples. *IC*_50_ (µg/mL) is defined as the concentration of an extract that causes 50% loss of the colour. The higher the radical scavenging potential is, the lower the value of *IC*_50_. The mean *IC*_50_ was calculated on the basis of three repetitions and by means of interpolation of the graphical dependence of concentration and degree of inhibition of the DPPH radical. Ascorbic acid and quercetin were used as standards.

Trolox equivalent antioxidant capacity (TEAC) was determined according to the procedure described by Brand-Williams et al. [95]. Briefly, 100 μL of the extract or Trolox standard was mixed with 2.9 mL of 0.12 mM DPPH (2,2-diphenyl-1-picrylhydrazyl) solution, prepared by dissolving 4.8 mg of DPPH in 100 mL of methanol (CH_3_OH). The mixtures were shaken and incubated for 30 min at room temperature in the dark. Absorbance was measured at 517 nm using a Camspec M508 spectrophotometer (Camspec Ltd., Leeds, UK). Antioxidant activity was quantified using a Trolox calibration curve (6-hydroxy-2,5,7,8-tetramethylchroman-2-carboxylic acid) in the concentration range of 0.045–1.5 mmol/L. The results were expressed as mmol Trolox equivalents (TE) per gram (mM TE/g).

#### 3.6.2. Hydrogen Peroxide Scavenging Activity (HPSA)

The hydrogen peroxide scavenging activity was evaluated according to the method described by Manolov et al. [96]. A 43 mM H_2_O_2_ solution was prepared in potassium phosphate buffer (0.2 M, pH 7.4). Briefly, 0.6 mL of H_2_O_2_ solution (43 mM), 1.0 mL of the sample or standard at different concentrations (20–1000 μg/mL), and 2.4 mL of potassium phosphate buffer were mixed in test tubes. The reaction mixtures were stirred and incubated in the dark for 10 min at 37 °C. Absorbance was measured at 230 nm using a Camspec M508 spectrophotometer (Camspec Ltd., Leeds, UK), against a blank containing phosphate buffer and H_2_O_2_ without the sample. Ascorbic acid and quercetin were used as standards. The percentage HPSA of the samples was evaluated by comparing with a blank sample and calculated using the following formula:

(2) $$I,\%\;\left(HPSA\right)=\;\left[\frac{A_{blank}-\left(A_{TS}-A_{CS}\right)}{A_{blank}}\right]\times 100\;$$

where *A_blank_* is the absorbance of the blank sample, *A_CS_* is the absorbance of the control sample, and *A_TS_* is the absorbance of the test sample.

#### 3.6.3. Hydroxyl Radical Scavenging Activity (HRSA)

The hydroxyl radical-scavenging activity of samples was measured according to the method of Luo et al. [97]. In this system, hydroxyl radicals were generated by the Fenton reaction. Hydroxyl radicals could oxidize Fe^2+^ into Fe^3+^, and only Fe^2+^ could be combined with 1,10-phenanthroline to form a red compound (1,10-phenanthroline-Fe^2+^) with the maximum absorbance at 536 nm. The concentration of hydroxyl radical was reflected by the degree of decolourization of the reaction solution. In test tubes, 1,10-phenanthroline solution (0.75 mM), 1 mL; phosphate-buffered saline (0.2 mol/L, pH 7.40), 2.0 mL; and 1 mL samples/standard were added and mixed homogeneously. Then, 1.0 mL of the FeSO_4_·7H_2_O solution (0.75 mM) was pipetted into the mixture. The reaction was initiated by adding 1.0 mL H_2_O_2_ (0.03% *v*/*v*). After incubation at 37 °C for 60 min in a water bath, the absorbance of the reaction mixture was measured at 536 nm against a reagent blank. The reaction mixture without any antioxidant was used as the negative control, and without H_2_O_2_ was used as the blank. The hydroxyl radical scavenging activity (HRSA) was calculated by the following formula:

(3) $$I,\%\;\left(HRSA\right)=\;\left[\frac{A_{S}-A_{n}}{A_{b}-A_{n}}\right]\times 100\;$$

where *A_S_*, *A_n_*, and *A_b_* were the absorbance values determined at 536 nm of the sample, the negative control, and the blank after reaction, respectively. Quercetin was used as a positive control.

### 3.7. Inhibition of Albumin Denaturation (IAD)

The in vitro anti-inflammatory activity was evaluated by measuring the inhibition of albumin denaturation (IAD). The assay was performed according to the method described by Manolov et al. [98], with minor modifications. Human serum albumin was used in the experiment, and a 1% (*w*/*v*) albumin solution was prepared in distilled water (pH 7.4). To avoid the potential effects of conventional organic solvents on albumin stability, green solvents were employed. Specifically, a natural deep eutectic solvent (NADES), urea:glycerol (1:2, molar ratio), was used [99]. Test samples and standards were initially dissolved in the NADES urea:glycerol (1:2) (U^1^G^2^) to obtain a stock solution with a final concentration of 1300 μg/mL. Subsequently, a series of working solutions with concentrations ranging from 5 to 1000 μg/mL was prepared in phosphate-buffered saline (PBS). The reaction mixture consisted of 2.0 mL of the test sample or standard at the appropriate concentration and 1.0 mL of albumin solution (1%). The mixtures were incubated at 37 °C for 15 min and then heated at 70 °C for 15 min in a water bath. After cooling to room temperature, turbidity was measured at 660 nm using a Camspec M508 spectrophotometer (Camspec Ltd., Leeds, UK). Ibuprofen was used as the reference standard. All experiments were performed in triplicate. The percentage inhibition of albumin denaturation (IAD) was calculated relative to the control. To assess the in vitro anti-inflammatory potential of *Dysphania ambrosioides* essential oil (DA-EO), its inhibitory effect was investigated. The mathematical description of the dose–response relationship was realized by applying nonlinear logistic models—four-parameter logistic (4PL) and five-parameter logistic (5PL) regressions [88,89,100], the adequacy of which was compared with the classical log-linear regression analysis (LR).

(4) $$4PL\;\;model\;\;\;\;\;\;\;\;y=A_{2}+\frac{A_{1}-A_{2}}{1+{\left(\frac{x}{{IC}_{50}}\right)}^{p}}\;$$

(5) $$5PL\;\;model\;\;\;\;\;\;\;\;y=A_{2}+\frac{A_{1}-A_{2}}{{\left[1+{\left(\frac{x}{{IC}_{50}}\right)}^{p}\right]}^{s}}\;$$

(6) R  model          y=a.lgx+b 

*A*_1_ and *A*_2_ represent the upper and lower asymptotes of the curve, ***p***—Hill slope parameter, ***s***—asymmetry, *a*—slope, and *b*—intercept.

### 3.8. Statistical Analysis

All of the analyses were performed in triplicate. The data were expressed as mean ± SD. ANOVA data analysis was performed using the statistical tool GraphPad Prism 10.4.0 and MS Office Excel software. The level of significance was fixed at *p* < 0.05. The mean *IC_50_* value was calculated from three replicates.

Statistical tests (Equations (7)–(10)) allow comparison of different models (4PL, 5PL, LR) for assessing the IAD of DA-EO with varying numbers of parameters, determining whether there is a statistically significant difference in model fit. The adjusted coefficient of determination ($R_{ad}^{2}$), Akaike information criterion (AIC), Bayesian information criterion (BIC), and residual sum of squares (RSS) were applied to the same set of experimental data [101,102],

(7) $$RSS=\sum_{i=1}^{n}{\left(y_{i}-{\hat{y}}_{i}\right)}^{2}\;$$

(8) $$AIC=n.ln\left(\frac{RSS}{n}\right)+2k\;$$

(9) $$BIC=n.ln\left(\frac{RSS}{n}\right)+k.ln\left(n\right)\;$$

(10) $$R_{ad}^{2}=1-\frac{RSS}{TSS}\;$$

where $y_{i}$ and ${\hat{y}}_{i}$ are the observed and predicted values of the model, respectively; *n*—number of observations; *k*—number of parameters; and *TSS*—total sum of squares.

## 4. Conclusions

The comprehensive study of the essential oil from *Dysphania ambrosioides* (DA-EO) originating from Bulgaria allows for fundamental conclusions regarding its phytochemical composition and biological potential. GC–MS analysis revealed a unique chemical profile dominated by oxygenated monoterpenes, classifying the studied sample as a specific thymol–carvacrol chemotype. The high concentrations of thymol and carvacrol distinguish this oil from the ascaridole-rich chemotypes reported in the literature and underlie its pronounced biological activity. The established phytochemical composition correlates directly with the demonstrated broad-spectrum antimicrobial effect against pathogenic microorganisms, positioning DA-EO as a promising natural agent for applications in the food and cosmetic industries. The antioxidant capacity of the oil, assessed using DPPH, HPSA, and HRSA assays, showed a clear dependence on the analytical method employed, with the highest efficacy observed in hydroxyl radical scavenging. Particularly notable are the findings on the anti-inflammatory potential, evaluated via the IAD assay. Application of precise nonlinear logistic models (4PL and 5PL) for *IC*_50_ calculation confirmed that the anti-inflammatory effect of DA-EO is comparable to that of standard nonsteroidal anti-inflammatory drugs, such as ibuprofen. Overall, the presented results contribute to a deeper understanding of the phytochemical variability of *D. ambrosioides* and provide a reliable foundation for future pharmacological investigations and the therapeutic utilization of the essential oil as a naturally derived bioactive product.

## Figures and Tables

**Table 1:** Chemical composition of DA-EO. Content of individual constituents calculated by normalization [%] and essential oil yield [% *v*/*w*]. RI denotes the retention index relative to *n*-alkanes (C_8_–C_40_) on a DB-5MS column; MS identification was performed by comparison of mass spectra with those contained in the NIST 08 mass spectral library. The third column with RI_L_ values is based on literature data (NIST’08 and Adams, 2007) [70], while the fourth column with RI_C_ contains the values calculated relative to a standard mixture of hydrocarbons (C_8_–C_40_, Sigma).

Peak	RT	RI_L_	RI_C_	Name	% of TIC
1	10.98	979	981	6-Methyl-5-hepten-2-one	0.09
2	11.14	990	989	6-Methyl-5-hepten-2-ol	0.10
3	11.96	1015	1014	α-Terpinene	0.94
4	12.24	1022	1020	*p*-Cymene	7.87
5	12.37	1025	1024	Limonene	1.15
6	13.28	1053	1054	γ-Terpinene	0.14
7	14.30	1087	1086	Terpinolene	0.19
8	14.64	1095	1095	β-Linalool	0.50
9	15.32	1120	1119	*trans*-*p*-2,8-Menthadien-1-ol	1.25
10	15.76	1130	1133	*cis*-*p*-2,8-Menthadien-1-ol	0.85
11	16.05	1142	1141	Camphor	2.72
12	16.87	1166	1165	Borneol	0.76
13	17.06	1173	1174	Terpinen-4-ol	0.40
14	17.31	1187	1186	α-Terpineol	2.44
15	17.52	1200	1199	γ-Terpineol	0.62
16	17.67	1206	1204	Verbenone	0.45
17	18.25	1214	1215	*trans*-Carveol	0.63
18	18.34	1227	1226	*cis*-Carveol	1.05
19	18.54	1231	1230	n-Hexyl 2-methylbutanoate	1.41
20	18.78	1234	1234	Ascaridole	0.98
21	18.94	1240	1239	Carvone	2.38
22	19.48	1245	1244	Car-3-en-2-one	0.58
23	20.12	1250	1249	Anethole	3.22
24	20.35	1291	1289	Thymol	19.45
25	20.60	1300	1298	Carvacrol	14.30
26	21.27	1328	1330	n-Hexyl tiglate	0.32
27	21.60	1336	1334	Methyl ο-anisate	0.71
28	21.74	1345	1346	α-Terpinyl acetate	0.45
29	21.91	1354	1356	Eugenol	1.45
30	22.41	1385	1385	β-Cubebene	0.24
31	22.49	1390	1389	β-Elemene	0.43
32	23.64	1415	1417	Caryophyllene	0.90
33	24.33	1452	1453	*cis*-Geranylacetone	0.76
34	25.13	1473	1474	Geranyl propanoate	1.56
35	25.18	1479	1480	α-Curcumene	1.74
36	25.39	1490	1489	β-Selinene	0.28
37	25.48	1496	1498	α-Selinene	3.95
38	25.84	1505	1505	β-Bisabolene	0.44
39	25.97	1512	1510	Anisyl propanoate	1.13
40	26.09	1515	1514	β-Curcumene	0.63
41	26.15	1518	1517	Myristicin	0.76
42	26.29	1530	1528	*trans*-Octenyl cyclopentanone	1.38
43	26.60	1543	1545	Selina-3,7(11)-diene	0.47
44	27.51	1572	1574	Germacrene D-4-ol	0.55
45	27.64	1584	1582	Caryophyllene oxide	1.48
46	28.57	1615	1613	*epi-α*-Cadinol	0.41
47	29.41	1647	1649	β-Eudesmol	0.87
48	29.48	1653	1652	α-Cadinol	1.63
49	29.67	1654	1662	7-*epi-*α-Eudesmol	2.19
50	33.30	1863	1860	*cis*,*cis*-Farnesyl acetone	7.23
51	34.63	1912	1911	5-*trans*,9-*trans*-Farnesyl acetone	0.74
52	35.78	1946	1945	Phytol	1.17
53	38.43	2120	2120	*trans*-Phytyl acetate	1.42

**Table 2:** Comparative analysis of the inhibitory effect of various concentrations of *Dysphania ambrosioides* essential oil (DA-EO) against Gram-positive and Gram-negative pathogens using the disc diffusion method (zones of inhibition, mm). Antimicrobial activity was evaluated by measuring the inhibition zones in millimetres (mm) following 48 h of incubation at 37 °C. Data are presented as mean ± SD (n = 3).

DA-EO mg/mL	*E. coli* ATCC 8739	*E. coli* ATCC 25922	*S. aureus* ATCC 6538	*S. aureus* ATCC 25923	*S. enterica ATCC13076*	*L. monocytogenes ATCC19115*
Pure EO	15.00 ± 0.71	10.00 ± 0.00	11.00 ± 0.71	28.00 ± 0.71	16.00 ± 0.71	17.00 ± 0.71
39.8	9.00 ± 0.00	-	-	13.00 ± 0.71	10.00 ± 0.71	-
1.9	-	-	-	-	-	-

**Table 3:** Antioxidant activity of *Dysphania ambrosioides* essential oil (DA-EO), evaluated by DPPH, HPSA, and HRSA methods. Data are presented as mean ± SD (n = 3). Ascorbic acid (AA) and quercetin (Qrc) were used as standards.

Samples	DPPH		HPSA	HRSA
	µM TE/g	*IC*_50_, µg/mL	*IC*_50_, µg/mL	
AA	-	3.19 ± 0.03	24.84 ± 0.35	-
Qrc	-	4.98 ± 0.33	69.25 ± 1.82	70.11 ± 1.17
DA-EO	24.71 ± 0.25	2572 ± 113	163 ± 4	97.55 ± 1.37

**Table 4:** Dose–response parameters and *IC*_50_ values for ibuprofen and DA-EO determined by inhibition of albumin denaturation (IAD) using linear regression (LR) and four- and five-parameter logistic models (4PL and 5PL). The data are presented as mean ± SD (n = 3). *A*_1_ and *A*_2_ represent the upper and lower asymptotes of the curve.

Parameters	4PL		5PL		LR	
	Ibuprofen	DA-EO	Ibuprofen	DA-EO	Ibuprofen	DA-EO
*A*_1_	0.963 ± 0.008	0.989 ± 0.019	0.961 ± 0.007	0.978 ± 0.016	-	-
*A*_2_	0.012 ± 0.001	0.043 ± 0.002	0.011 ± 0.001	0.049 ± 0.004	-	-
*IC*_50_, µg/mL	56.9 ± 1.2	67.0 ± 1.7	64 ± 6	77 ± 7	60.7 ± 1.1	83 ± 5
Hill slope (***p***)	3.23 ± 0.09	2.95 ± 0.21	3.25 ± 0.22	3.10 ± 0.15	-	-
***s***	-	-	1.29 ± 0.15	1.33 ± 0.23	-	-
R^2^	0.9988	0.9967	0.9992	0.9992	0.8802	0.8868

**Table 5:** Statistical comparison of dose–response models (4PL, 5PL, and LR) for ibuprofen and *Dysphania ambrosioides* essential oil (DA-EO) based on residual sum of squares (RSS), Akaike information criterion (AIC), Bayesian information criterion (BIC), and adjusted coefficient of determination ($R_{ad}^{2}$) values obtained from the albumin denaturation assay.

Statistical Parameters	4PL		5PL		LR	
	Ibuprofen	DA-EO	Ibuprofen	DA-EO	Ibuprofen	DA-EO
RSS	1.80 × 10^−3^	3.40 × 10^−3^	1.60 × 10^−3^	3.30 × 10^−3^	0.164	0.163
AIC	−59.20	−54.11	−58.14	−52.35	−27.10	−27.15
BIC	−58.88	−53.79	−57.74	−51.95	−26.94	−26.99
$R_{ad}^{2}$	0.9987	0.9974	0.9988	0.9974	0.9038	0.8740

## Data Availability

Data are contained within the article.

## Supplementary Materials

The following supporting information can be downloaded at: https://www.mdpi.com/article/10.3390/molecules31060946/s1, Figure S1: Chromatographic profile of *Dysphania ambrosioides* essential oil.

## Abbreviations

DA-EO	*Dysphania ambrosioides* essential oil
EO	Essential oil
4PL	Four-parameter logistic regression
5PL	Five-parameter logistic regression
LR	log-linear regression
AIC	Akaike Information Criterion
BIC	Bayesian Information Criterion
RSS	Residual Sum of Squares
$R_{ad}^{2}$	Adjusted coefficient of determination
DPPH	2,2-diphenyl-1-picrylhydrazyl
HPSA	Hydrogen peroxide scavenging activity
HRSA	Hydroxyl radical scavenging activity
IAD	Inhibition of albumin denaturation
AA	Ascorbic acid
Qrc	Quercetin
