# Supplementary material for: Chemical Composition and Biological Activity of Essential Oil from Dysphania ambrosioides from Bulgaria
Source: Molecules. 2026 Mar 12;31(6):946. doi: 10.3390/molecules31060946 (PMC13028757; doi:10.3390/molecules31060946)
Supplement: Supplementary file 1 [file molecules-31-00946-s001.zip › Supplementari Material.pdf]

Chemical Composition and Biological Activity of Essential Oil From *Dysphania ambrosioides* From Bulgaria

Andjelika Nacheva[1], Dimitar Bojilov[1*], Stanimir Manolov[1], Iliyan Ivanov[1], Soleya Dagnon[1], Ivayla Dincheva[2], Neli Grozeva[3], Bogdan Goranov[4], and Zlatka Ganeva[4]

Contents

Figure S1. Chromatographic profile of *Dysphania ambrosioides* essential oil. 1

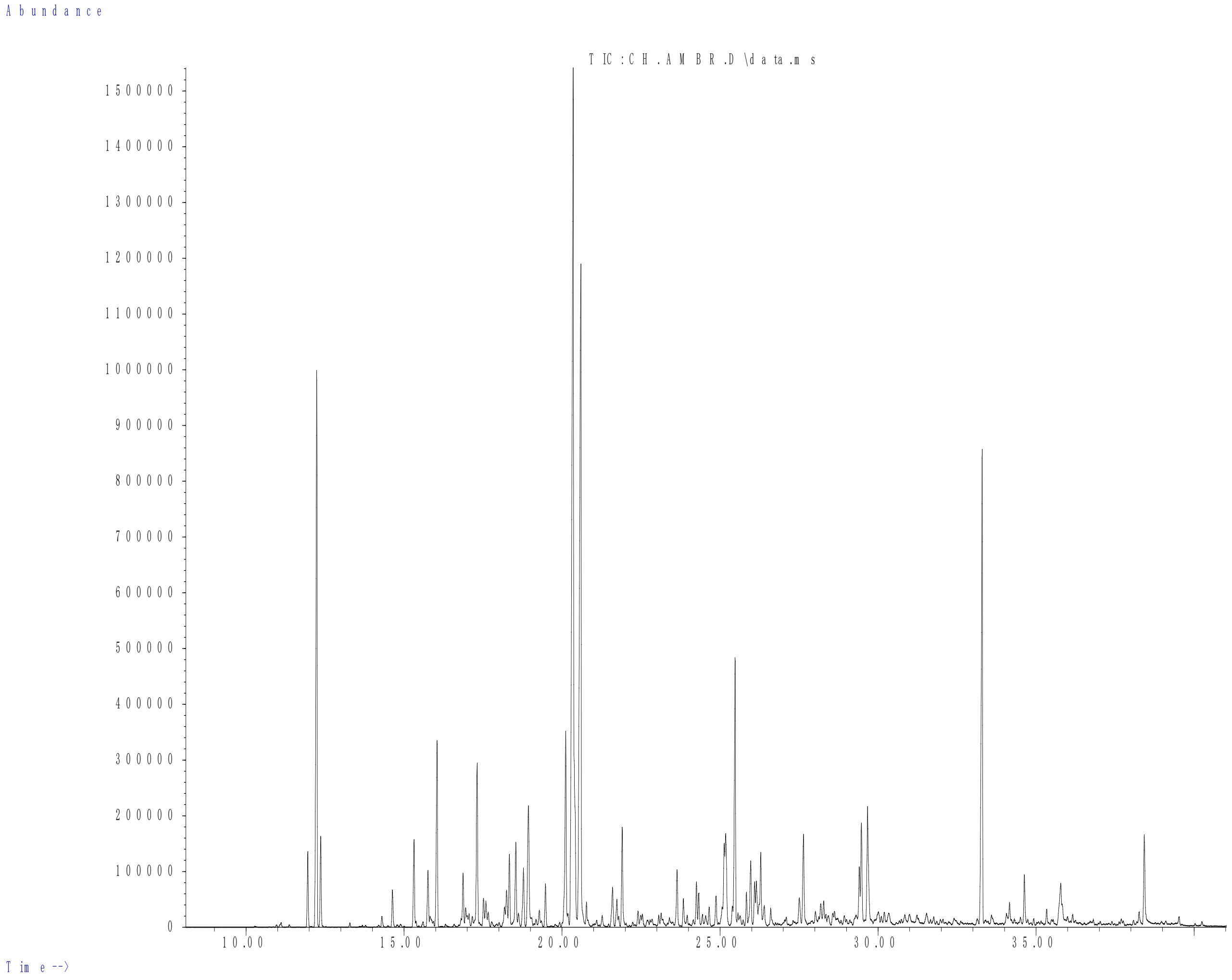

Figure S1. Chromatographic profile of *Dysphania ambrosioides* essential oil.
